# Early detection of uterine corpus endometrial carcinoma utilizing plasma cfDNA fragmentomics

**DOI:** 10.1186/s12916-024-03531-8

**Published:** 2024-07-29

**Authors:** Jing Liu, Dan Hu, Yibin Lin, Xiaoxi Chen, Ruowei Yang, Li Li, Yanyan Zhan, Hua Bao, LeLe Zang, Mingxuan Zhu, Fei Zhu, Junrong Yan, Dongqin Zhu, Huiqi Zhang, Benhua Xu, Qin Xu

**Affiliations:** 1 Department of Gynecology, Clinical Oncology School of Fujian Medical University, Fujian Cancer Hospital (Fujian Branch of Fudan University Shanghai Cancer Center), Fuzhou, China; 2 Department of Pathology, Clinical Oncology School of Fujian Medical University, Fujian Cancer Hospital (Fujian Branch of Fudan University Shanghai Cancer Center), Fuzhou, China; 3 Geneseeq Research Institute, Nanjing Geneseeq Technology Inc., Nanjing, Jiangsu China; 4 Department of Radiation, Fujian Medical University Union Hospital, Fuzhou, China

**Keywords:** Early detection, Endometrial carcinoma, cfDNA, Fragmentomics

## Abstract

**Background:**

Uterine corpus endometrial carcinoma (UCEC) is a prevalent gynecologic malignancy with a favorable prognosis if detected early. However, there is a lack of accurate and reliable early detection tests for UCEC. This study aims to develop a precise and non-invasive diagnostic method for UCEC using circulating cell-free DNA (cfDNA) fragmentomics.

**Methods:**

Peripheral blood samples were collected from all participants, and cfDNA was extracted for analysis. Low-coverage whole-genome sequencing was performed to obtain cfDNA fragmentomics data. A robust machine learning model was developed using these features to differentiate between UCEC and healthy conditions.

**Results:**

The cfDNA fragmentomics-based model showed high predictive power for UCEC detection in training (*n* = 133; AUC 0.991) and validation cohorts (*n* = 89; AUC 0.994). The model manifested a specificity of 95.5% and a sensitivity of 98.5% in the training cohort, and a specificity of 95.5% and a sensitivity of 97.8% in the validation cohort. Physiological variables and preanalytical procedures had no significant impact on the classifier’s outcomes. In terms of clinical benefit, our model would identify 99% of Chinese UCEC patients at stage I, compared to 21% under standard care, potentially raising the 5-year survival rate from 84 to 95%.

**Conclusion:**

This study presents a novel approach for the early detection of UCEC using cfDNA fragmentomics and machine learning showing promising sensitivity and specificity. Using this model in clinical practice could significantly improve UCEC management and control, enabling early intervention and better patient outcomes. Further optimization and validation of this approach are warranted to establish its clinical utility.

**Supplementary Information:**

The online version contains supplementary material available at 10.1186/s12916-024-03531-8.

## Background

Uterine corpus endometrial carcinoma (UCEC) is a prevalent gynecologic malignancy and a major cause of cancer-related morbidity in women [1]. It is generally perceived that UCEC has a favorable prognosis during the early stage. The 5-year survival rate for patients diagnosed with stage I/II endometrial carcinoma is typically between 82 and 90%, whereas for those diagnosed at stage III/IV, the rate significantly drops to between 34 and 42% [2]. Consequently, the importance of accurate initial diagnosis and prompt treatment are key in the management of endometrial carcinoma. Early detection of UCEC allows for the utilization of minimally invasive surgical techniques and diminishes the necessity for adjuvant treatments, thereby lowering healthcare costs, morbidity, and mortality rates [3, 4]. However, there is currently no sufficiently accurate and reliable early detection test for UCEC that can be applied to high-risk women with suspected UCEC. Transvaginal ultrasonography (TVU), the most commonly employed method, exhibits high sensitivity in detecting UCEC in postmenopausal women with abnormal uterine bleeding. However, its relatively low specificity necessitates further tests to definitively rule out endometrial malignancy [5]. Conversely, while hysteroscopy provides enhanced precision, its invasive character can lead to complications or patient-related issues, thereby contributing to its potential failure [6]. Therefore, the development of a precise, non-invasive diagnostic method for endometrial carcinoma is of great importance.

Peripheral blood collection, a minimally invasive procedure, has surfaced as a novel method for the early detection of a wide array of solid malignancies, particularly by providing genetic information of circulating tumor markers [7]. At present, several studies are analyzing circulating tumor DNA (ctDNA) and microRNAs for the early detection of endometrial carcinoma [8, 9]. However, with regard to early diagnosis of endometrial carcinoma, the technology to diagnose this condition solely through gene sequencing of circulating tumor components in the blood is yet to be fully developed and optimized.

Currently, plasma cfDNA fragmentomics has been applied to numerous solid malignancies for enhanced diagnostic precision [10–12]. A significant advancement was made with the development of DNA Evaluation of Fragments for Early Interception (DELFI) [13], which assesses fragment coverage, size, and other summary statistics within 5 Mb windows. It was observed that the ratio of short to long cfDNA fragments within each window varies between cancer patients and healthy individuals, thereby providing potential discrimination of disease status. Studies in fragmentomics have also leveraged other distinguishing patterns between cancer and non-cancer groups, such as preferred end coordinates and end motifs [14, 15]. Recently, there has been a research focus on the nucleosome footprint of cfDNA, with successful identification of patient-specific and tumor-specific patterns with substantial accuracy [16, 17].

In the present study, we developed a robust machine learning model that utilizes low-coverage whole-genome sequencing and an extensive set of genome-wide features derived from cfDNA fragmentomics to differentiate between UCEC and healthy conditions. The primary objective of this research is to provide a highly sensitive and cost-effective model for UCEC detection, which could potentially yield substantial clinical advantages in the management and control of UCEC.

## Methods

### Patients and sample collection

In this study, a total of 121 patients with UCEC and 119 healthy female donors were initially enrolled at the Fujian Cancer Hospital (Fujian Branch of Fudan University Shanghai Cancer Center) from August 2021 to July 2022 and from November 2022 to February 2023 (Additional file 1: Fig. S1). Participants were subsequently excluded if they were lost to follow-up, had post-treatment status, withdrew their consent, or failed to meet quality control criteria. After the exclusions, the study proceeded with 111 UCEC patients and 111 healthy donors for further analysis. A minimum collection volume of 30 ml of peripheral blood samples was collected from patients before any treatment or healthy donors and proceeded for low-depth whole-genome sequencing (WGS). One participant was excluded for failed NGS quality control. All healthy control participants underwent a thorough physical examination and were monitored for a period of one year, with routine follow-up conducted every three months to promptly identify any onset of cancer. A training cohort and an independent validation cohort were established separately based on time of enrollment. The training cohort was dedicated to train a multi-dimensional machine learning model, whereas the independent validation cohort set out to assess the performance of the model. An additional cohort of 47 patients with hysteromyoma was retrospectively collected to further validate our findings and assess the generalizability of our model across diverse patient populations. The genetic tests were performed in a centralized clinical testing center (Nanjing Geneseeq Technology Inc., China; Certified to CAP, CLIA, and ISO15189). The study was performed in accordance with the Declaration of Helsinki and approved by the Ethics Committee of Fujian Cancer Hospital. All patients provided oral and written informed consent to participation and publication.

### Next-generation sequencing and data processing

Blood samples, drawn into EDTA tubes, were centrifuged at 16,000 × for 10 min within 4 h post-collection. The QIAamp Circulating Nucleic Acid Kit (Qiagen) was used to extract cfDNA from the plasma samples, without the inclusion of carrier RNA, following the manufacturer’s instructions. To ensure sufficient cfDNA for further analysis, the concentration of cfDNA in the plasma was determined using the Qubit dsDNA HS Assay Kit (Thermo Fisher Scientific) according to the manufacturer’s guidelines.

The extracted plasma cfDNA was then used for WGS with the KAPA Hyper Prep Kit (KAPA Biosystems), following the manufacturer’s instructions. Briefly, 5–10 ng of cfDNA per sample underwent a series of steps including end-repair, A-tailing, and ligation with adapters. The resulting libraries were quantified using the KAPA SYBR FAST qPCR Master Mix (KAPA Biosystems), and then loaded onto NovaSeq platforms (Illumina) for paired-end sequencing, as recommended by the manufacturer.

To ensure data quality, the sequencing output was subjected to quality control measures. Trimmomatic [18] was used for read trimming, followed by PCR duplicate removal using Picard tools (Broad Institute, MA, USA). The trimmed reads were aligned to the human reference genome (GRCh37/UCSC hg19) using the Burrows-Wheeler Aligner [19]. The median coverage depth across all samples was 8.17 × (Additional file 2). To standardize the data and mitigate the effects of variable sequencing depth, we applied a down-sampling procedure. Coverage depths that exceeded 5 × were reduced to a uniform 5 × , ensuring consistency across these samples. For samples that originally had lower coverage, no down-sampling was performed; they were analyzed at their initial sequencing depths.

### Genome-wide cfDNA features

To construct a multi-facet machine learning model that differentiates UCEC and healthy individuals, fragmentomics and CNV features were extracted from the processed WGS data using an in-house script. Copy number variation (CNV), fragment size distribution (FSD), and nucleosome footprint (NF) profiling were used to construct the final model (Additional file 3).

The profiling of copy number variations was adapted from the method as described by Wan et al. [20]. Each sample’s genome was partitioned into bins of 1 Mb, resulting in a total of 2475 bins. A Hidden Markov Model was employed to compare the depth of each bin with the software baseline, generating a log2 ratio for each bin. This analysis allowed for the identification of copy number variations across the genome.

The fragment size distribution (FSD) feature quantifies the coverage of cfDNA fragments ranging from 110 to 220 bps in 5-bp stepwise (e.g., 110–114 bps, 115–119 bps, …, 215–220 bps; 24 bins) at every chromosome arm. This creates a detailed series of 24 bins across representing the distribution of cfDNA fragment size of each chromosome arm. A total of 39 chromosome arms are resolved into 936 discrete FSD features, contributing to the granularity of our analysis. The short arms of 5 acrocentric chromosomes were not included as they remained largely unsequenced to date. To ensure a focus on intrinsic, biologically relevant patterns rather than variations introduced by whole genome sequencing, we standardized the raw FSD coverage values by converting them into *z*-scores. This normalization contrasts each value against the overall mean within each sample. This approach enabled the enhanced detection of high-resolution chromosome-level patterns, which could potentially reveal further distinctions between the cancer and non-cancer groups.

For nucleosome footprint (NF) profiling, the framework developed by Doebley et al. [17] was utilized to analyze nucleosome occupancy in cfDNA while accounting for GC bias. We quantified the GC-corrected coverage profile using three observable characteristics: the central coverage value 30 bp away from the location, the “average coverage” value within a 1000-bp distance from the location, and the amplitude determined through fast-Fourier yransform. In this study, we analyzed a total of 854 transcription factor binding sites to optimize the ability to detect cancer from low-pass WGS data.

### Model construction and cross-validation analyses

We developed a two-tiered machine learning framework to differentiate between cancerous and non-cancerous samples. The initial tier of our framework comprised a feature-specific module that processed one of three distinct feature sets: copy number variations (CNV), feature selection dimensionality (FSD), or nuclear features (NF). This module systematically applied a suite of five foundational algorithms, namely Generalized Linear Model (GLM), Gradient Boosting Machine (GBM), Distributed Random Forest (DRF), Deep Learning (DL), and XGBoost. A grid search methodology was employed to optimize the hyperparameters for each algorithm, drawing from predefined candidate values. We assessed the efficacy of these models using a fivefold cross-validation scheme, ensuring consistency in fold assignment across all feature types for both training and validation.

In the second phase of our analysis, the ensemble model of each feature type was constructed by averaging the outputs of the five leading models (determined by their cross-validation area under the curve (AUC) scores) from each category. For incoming samples, the final classification score was computed as the average prediction score from these three top-tier ensemble models, with the score ranging between 0 and 1—where a higher score indicated an increased probability of the sample being cancerous.

In the training cohort, our classifiers underwent a fivefold cross-validation, aiming for a specificity threshold of 95% to set the model’s decision boundary. An independent validation cohort was subsequently employed to assess the efficacy of our composite machine learning model.

To assess the stability and generalizability of the ensemble model, we employed a method of repeated random partitioning of the cohort. The entire dataset was randomly split into three distinct sets: a training set, a validation set, and a test set. This random partitioning was not a single event but was repeated multiple times to produce a comprehensive range of data subsets, thereby simulating a variety of potential training and testing scenarios. The training set was used to build the model, the validation set to tune the hyperparameters, and the test set to evaluate the model’s performance. This approach mitigated the risk of overfitting and provided a more robust estimation of the model’s performance in unseen data.

### Tumor fraction calculation

To quantify the proportion of ctDNA within the cell-free DNA (cfDNA) and to evaluate copy number variations (CNVs) characteristic of tumor-derived DNA, we utilized the computational tool ichorCNA[20]., which is designed to work with high-throughput sequencing data. Each sample’s genome was partitioned into bins of 1 Mb, resulting in a total of 2475 bins. A Hidden Markov Model was employed to compare the depth of each bin with the software baseline, generating a log2 ratio for each bin. This analysis allowed for the identification of copy number variations across the genome. The model also integrates several data features, including total read depth, B-allele frequency, and the distribution of cfDNA fragment lengths. Through this integrated analysis, ichorCNA provides estimates of the tumor fraction, defined as the proportion of cfDNA attributable to tumor cells. The clinical limit of detection was further calculated following the approach described by Jamshidi et al. [21], defining it as the tumor fraction corresponding to a 50% probability of detecting a cancer signal.

### Nucleosome footprint differentiation analysis and corresponding gene-enriched pathways

To ascertain nucleosome footprint (NF) features that exhibited unique signatures in uterine corpus endometrial carcinoma (UCEC) relative to healthy individuals, we engaged in a comparative study of NF profiles from both cohorts. Employing the multiple t-tests, we pinpointed NF features with significant variances in central coverage, average coverage, and amplitude. Features with adjusted *p*-value < 0.01 (Benjamini–Hochberg method) were retained for further analysis. These selected features were presumed to be reflective of UCEC-specific chromatin organization. Subsequently, we mapped the genes associated with these NF features to their respective biological pathways via the Encyclopedia of Genes and Genomes (KEGG) pathway analysis. We then rigorously evaluated the connections between these genes and their related biological functions and pathways, adopting a significance cutoff (*P* < 0.05) to identify pathways potentially implicated in the pathogenesis or progression of UCEC.

### Pre-analytical and physiological variables analysis

To evaluate the potential effect of various pre-analytical variables and physiological variables on model robustness, a subset of true positive or true negative participants, specifically 21 healthy participants and 4 UCEC patients in the validation cohort were further analyzed. Blood samples were collected and tested multiple times to assess consistency and reproducibility, with the Positive Percent Agreement (PPA) calculated for each set of results. The impact of different transportation (24, 48, and 72 h post-collection) and storage conditions (room temperature and with an ice pack) were examined, with the reference condition defined as the state of the sample within 2 h of collection. The effect of freezing duration of plasma (3 days, 7 days, 1 month, and 6 months) on the test outcomes was also studied. Additionally, the influence of different physiological states, specifically before and after meals and exercise, was also evaluated by repeated blood collection. For each set of conditions, the PPA was calculated, and a 95% confidence interval was computed, using the stability of the PPA under different conditions to gauge the robustness and reliability of the test outcomes.

### Clinical benefit analysis

To evaluate the potential clinical advantages of our model in practical settings, we employed a methodology proposed by Hubbell et al. [22], which involved integrating their interception model with the predictions generated in our current study. This approach was applied to assess the impact on colorectal cancer incidence in a Chinese cohort.

### Statistical analysis

Multivariate analysis was performed with clinical variables that were statistically significant in univariate analysis. All *P* values were based on two-sided testing unless specified, and differences were considered significant at *P* < 0.05. For the calculation of 95% confidence intervals (CIs) for sensitivity and specificity, we applied Wilson’s score interval formula. To estimate the 95% CI for the AUC, we employed the bootstrap resampling technique. Specifically, 1000 bootstrap samples were generated from the validation dataset, and the AUC was recalculated for each sample to create an empirical distribution of AUC values. The 95% CI was then determined by identifying the 2.5th and 97.5th percentiles of this distribution. Positive Percent Agreement (PPA) is measured between the agreement between the results of optimal condition and test condition. Statistical analysis was performed using R software, version 4.2.3.

## Results

### Patient characteristics

**Fig. 1:**
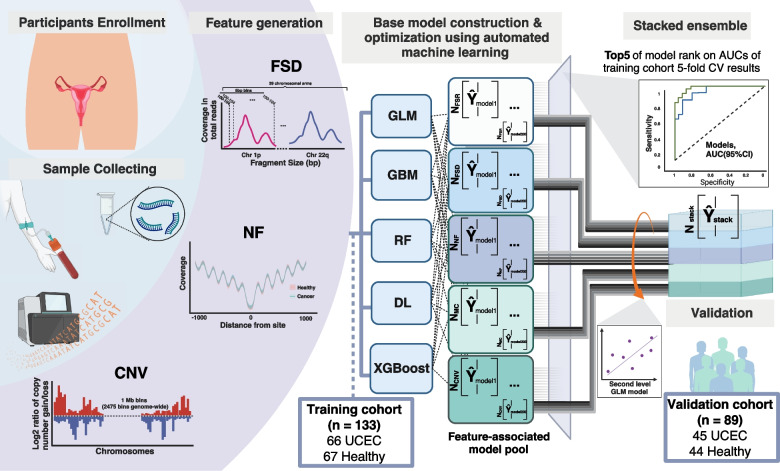
Schematic diagram illustrating the study design

A total of 111 UCEC and 111 healthy individuals were included in the study. The demographic and clinical characteristics of all participants were provided in Additional file 4. The training cohort dataset, comprising 66 UCEC patients and 67 healthy individuals, was exclusively used for training the model. To validate the model, an independent cohort was utilized, which was distinct from the training dataset in terms of temporal recruitment. This validation cohort consisted of 89 participants, including 44 UCEC patients and 45 healthy individuals (Fig. 1; Additional file 1: Fig. S1).

The mean age for the UCEC patients was 54.5 years in the training cohort and 56.7 years in the validation cohort, compared to the mean age of healthy participants as 57.3 years in the training cohort and 51.5 years in the validation cohort (Table 1). The distribution of cancer stages was similar across both cohorts, with the majority of participants being in stage I. In the training cohort, 77.3% (*n* = 51) of participants were in stage I, while in the validation cohort, 60.0% (*n* = 27) were in stage I. In the context of histological grade, a substantial proportion of patients in both cohorts were classified as low risk, encompassing both Grade 1 and Grade 2. Specifically, in the training cohort, 81.8% (*n* = 54) of the patients were categorized as low risk, while the validation cohort had 71.1% (*n* = 32).

**Table 1:** Patient characteristic

	**All**		**Train**		**Valid**	
	**Healthy**	**UCEC**	**Healthy**	**UCEC**	**Healthy**	**UCEC**
	(***N*=111)**	(***N*=111)**	(***N*=67)**	(***N*=66)**	(***N*=44)**	(***N*=45)**
**Age**						
Mean [Min, Max]	56.0 [44, 72]	55.4 [33, 76]	57.3 [45, 72]	54.5 [33, 69]	51.5 [44, 67]	56.7 [35, 76]
**Stage**						
I (%)	-	78 (70.3%)	-	51 (77.3%)	-	27 (60.0%)
II	-	17 (15.3%)	-	9 (13.6%)	-	8 (17.8%)
III	-	10 (9.0%)	-	4 (6.1%)	-	6 (13.3%)
IV	-	4 (3.6%)	-	2 (3.0%)	-	2 (4.4%)
Not available	-	2 (1.8%)	-	0 (0%)	-	2 (4.4%)
**Grade**						
G1	-	35 (31.5%)	-	26 (39.4%)	-	9 (20.0%)
G2	-	51 (45.9%)	-	28 (42.4%)	-	23 (51.1%)
G3	-	16 (14.4%)	-	8 (12.1%)	-	8 (17.8%)
Not available	-	9 (8.1%)	-	4 (6.1%)	-	5 (11.1%)
**MSI status**						
dMMR/MSI-H	-	24 (21.6%)	-	41 (62.1%)	-	26 (57.8%)
MSS	-	67 (60.4%)	-	12 (18.2%)	-	12 (26.7%)
Not available		20 (18.0%)		13 (19.7%)		7 (15.5%)

### UCEC detection classifier performance

**Fig. 2:**
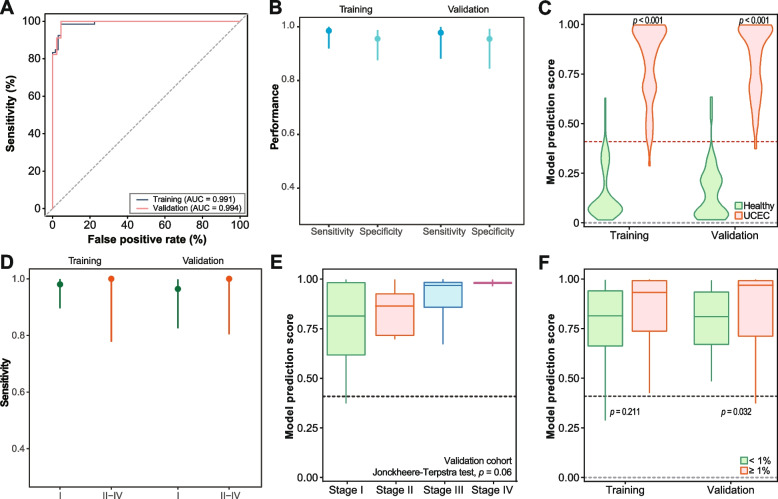
**A** ROC curve of ensemble stacked model of training and independent validation datasets. **B** Validation ROC curve of base models constructed upon individual features (CNV, FSD, and NF) and their ensemble stacked model. **C** Violin plots illustrating ensemble stacked model predicted cancer score distribution in the UCEC and healthy groups in the training and validation cohorts. **D** Sensitivity of the classifier in identifying cancer patients at different stages. Error bar represented 95% confidence intervals. **E** Boxplot showing the correlation between cancer scores and advancing stages. **F** Boxplot comparing cancer scores in samples with different tumor fraction. Tumor fraction was measured utilizing ichorCNA

The constructed model demonstrated excellent prediction power, with an area under the curve (AUC) of 0.991 (95% CI (0.9788, 0.999)) in the training cohort. This model was then validated in the independent cohort, where it achieved an AUC of 0.994 (95% CI (0.9810, 1.000)) (Fig. 2A). To highlight the superiority of the ensemble model, the base model using individual features generated AUCs ranging from 0.881 to 0.986 (Additional file 1: Fig. S2). Moreover, the stacked model demonstrated enhanced robustness, maintaining its performance with lower sequencing coverage depth (Additional file 1: Fig S3). This demonstrates that the ensemble model outperformed the base model in terms of predictive power. As a cancer classifier, a specific threshold of 0.423 was applied to the cancer scores to target a specificity of 95.0% in the training dataset. The established threshold maintained specificities of 95.5% (95% CI (87.5%, 98.8%)) within the training cohort and 95.5% (95% CI (84.4%, 99.2%)) within the validation cohort (Fig. 2B). The corresponding sensitivities were 98.5% (95% CI (91.9%, 99.9%)) in the training cohort and 97.8% (95% CI (88.2%, 99.9%)) in the validation cohort. The stacked model exhibited robust performance with a mean sensitivity of 0.97 (95% CI: 0.96, 0.98) and a mean specificity of 0.96 (95% CI: 0.94, 0.97) across multiple repeated random cohort splits (Additional file 1: Fig. S4), indicating stable and reliable diagnostic accuracy.

Furthermore, cancer patients had significantly higher predicted cancer scores compared to healthy individuals (*p* < 0.0001; Fig. 2C), further supporting the superior prediction power of the ensemble model, which incorporated multiple cfDNA fragmentomics features. Additionally, a retrospective analysis performed on a separate cohort of 47 patients diagnosed with hysteromyoma revealed that the model retained a commendable accuracy of 91.5% (95% CI: 80.1–96.6%; Additional file 1: Fig. S5).

We conducted further analysis in the validation cohort to assess the model’s performance across various UCEC subgroups. Notably, the classifier demonstrated high sensitivity, accurately identifying cancer patients with a sensitivity of 96.4% (95% CI (82.5%, 99.8%)) at the early stage (stage I) and maintained a sensitivity of 100% in later stage (Fig. 2D). Moreover, the cancer scores exhibited an upward trend in correlation with advancing stages (*p* = 0.06; Jonckheere Terpstra test; Fig. 2E). This trend is likely attributable to the higher tumor fractions typically present in later-stage disease (Additional file 1: Fig. S6A). In line with this, there was a significantly positive correlation between the cancer scores and the tumor fraction (Additional file 1: Fig. S6B). Samples with a tumor fraction exceeding 1% yielded significantly higher cancer scores compared to those with a lower tumor fraction (Fig. 2F). This observation highlights the biological relevance reflected by the cancer scores, suggesting that they may indeed capture the extent of tumor-derived cfDNA present in the plasma. Nevertheless, there were no significant differences observed in cancer scores among samples with varying histological grades or MSI status (Additional file 1: Fig. S7).

### Biological significance of Fragmentomics pattern in UCEC cancer

**Fig. 3:**
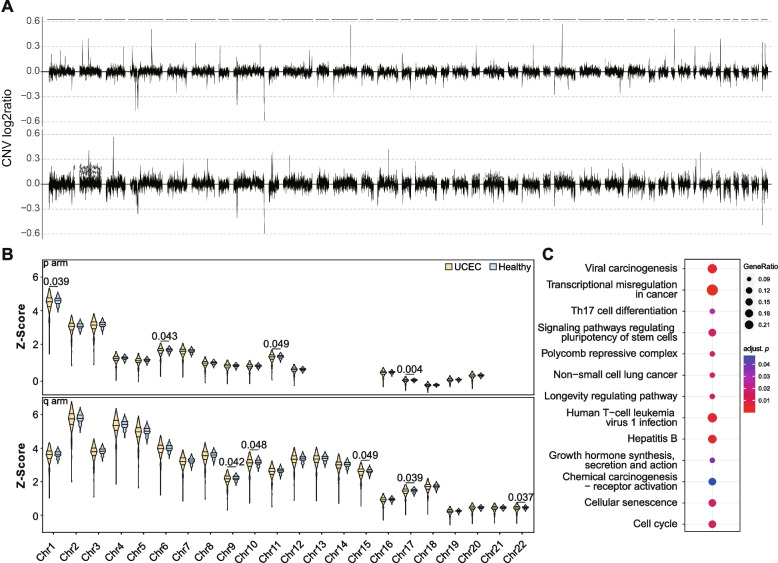
**A** The ratio of CNV in 1 Mb bins across the genome in UCEC patients and healthy controls. **B** Violin plots showing the difference of FSD distribution of each chromosome arm in UCEC and healthy participants. **C** KEGG pathway enrichment analysis of genes corresponding to NF features that displayed distinct characteristics in UCEC patients versus healthy controls

Furthermore, we investigate the biological implications of fragmentomics patterns in UCEC cancer. We observed genome-wide variation in Copy Number Variation (CNV) features (Fig. 3A; Additional file 1: Fig. S8A). A noteworthy proportion of these CNV features exhibited statistically significant differences (adjusted *P*-value < 0.05; Additional file 1: Fig. S9A). Our fragment size distribution (FSD) analysis revealed fragment length patterns with remarkable resolution (Additional file 1: Fig. S8B), even at the level of chromosomal arms. The FSD features pointed to a significant differential distribution across chromosome arm (Additional file 1: Fig. S10), with notable alterations in chromosomes 1, 10, 6, 11, and 17 (Fig. 3B). These chromosomes intriguingly align with five of the top ten chromosomes with the highest mutation frequencies in the TCGA-UCEC database (Additional file 1: Fig. S9B). Such a correlation hints at a potential interplay between disrupted nucleosome organization and mutation-prone genomic regions in UCEC.

Moreover, a total of 104 NF features that displayed distinct characteristics in UCEC patients versus healthy controls were selected (Additional file 5). The genes corresponding to these features were subjected to KEGG pathway enrichment analysis (Fig. 3C). A number of pathways related to cancer and immune response, such as transcription misregulation in cancer, viral or chemical carcinogenesis, and Th17 cell differentiation, were identified in the UCEC patients in comparison to healthy participants. Intriguingly, the enrichment of pathways related to cancer and immune responses was consistent across various stages of UCEC (Additional file 1: Fig. S9C), indicating that certain key pathogenic processes are persistently disrupted irrespective of disease progression. These findings underscore the biological relevance of the NF features used in our study, suggesting that the fragmentomics patterns identified are not merely numerical abstractions but are deeply rooted in the complex biology underlying cancer development and progression.

### Evaluation of preanalytical and physiological variables on cancer detection

**Fig. 4:**
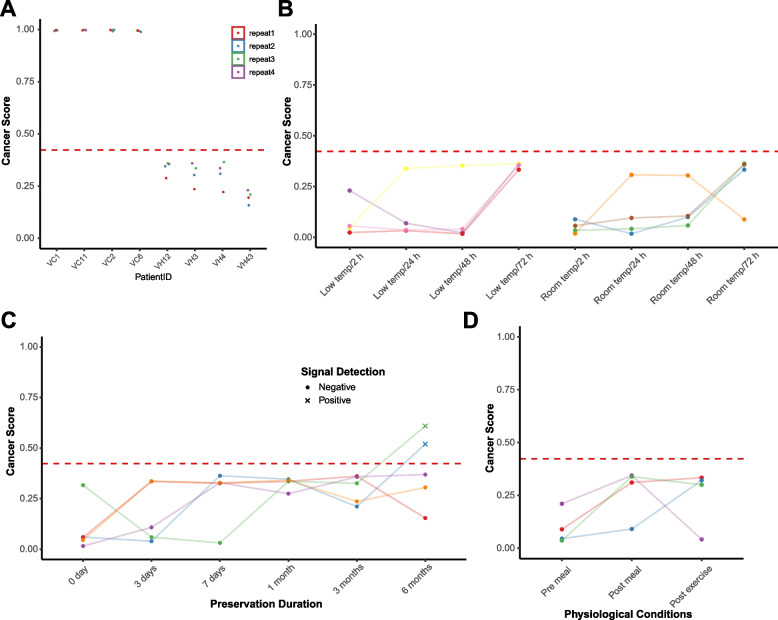
**A** The repeated test score of UCEC and healthy participants. **B** The impact of various transportation conditions on the test outcomes for healthy participants. **C** The impact of frozen durations on the test outcomes for healthy participants. **D** The impact of physiological conditions (states before and after meals as well as before and after exercise) on the test outcomes

The influence of physiological variables during blood collection and preanalytical procedures on the outcomes of the classifier was also assessed. Repeated blood collection from both the healthy and cancer-affected participants consistently yielded reproducible results. The PPA was remarkably stable, registering at 100.0% with a 95% confidence interval statistically ranging between 67.6% and 100.0% (Fig. 4A). In the case of the healthy participants, the transportation conditions of the samples—whether they were transported within 24 h, 48 h, or 72 h, and regardless of whether they were stored at room temperature or with an ice pack—did not affect the test outcomes. All samples consistently aligned with the reference condition, defined as the state within 2 h of collection (Fig. 4B). This was reflected in a PPA of 100.0%, with a 95% confidence interval ranging from 51.0 to 100.0%. Furthermore, most samples that were frozen for various durations (3 days, 7 days, 1 month) exhibited the same level of agreement with the reference condition as non-frozen samples, with a PPA of 100.0% and a 95% confidence interval ranging from 56.6 to 100.0%. However, samples frozen for 6 months deviated from this pattern, showing a higher risk score and thereby becoming a positive signal. The PPA for these samples was 60.0%, with a 95% confidence interval ranging from 23.1 to 88.2% (Fig. 4C). Physiological conditions, including states before and after meals as well as before and after exercise, did not influence the test outcomes. All samples under these conditions remained in agreement with the reference condition, with a PPA of 100.0% and a 95% confidence interval ranging from 51 to 100% (Fig. 4D). This suggests a high level of robustness and reliability in the test outcomes, irrespective of various physiological and preanalytical conditions.

## Discussion

In this study, we first developed and evaluated a classifier for the detection of UCEC using cfDNA fragmentomics features. The constructed ensemble model demonstrated excellent predictive power, as evidenced by high AUC values in both the training and independent validation cohorts. The AUCs achieved by the ensemble model were significantly higher than those of the individual base models, indicating the superiority of the ensemble approach in terms of predictive performance. The established cut-off value for the cancer scores yielded high specificities in both the training and validation cohorts, while maintaining high sensitivities. This suggests that the classifier utilizing cfDNA fragmentomics features can efficiently differentiate between cancer patients and healthy subjects with a minimal false-positive rate, accomplishing a significantly higher specificity than conventional transvaginal ultrasonography [5], and a superior sensitivity than circulation tumor DNA methodologies [23, 24]. The significantly higher cancer scores observed in UCEC patients compared to healthy individuals further support the robustness and accuracy of the classifier. Importantly, the classifier demonstrated remarkable sensitivity in detecting UCEC at early stages, with a sensitivity of 96.4% in stage I patients. Additionally, the prediction of the stage shift in UCEC diagnosis revealed that our model could identify approximately 20% of UCEC patients, who would otherwise be diagnosed at a more advanced stage (stage III/IV) under conventional care, at an earlier stage. This could result in enhanced 5-year survival rates, underscoring the classifier’s clinical utility in expediting diagnosis and intervention.

The fragmentomics patterns employed as features are not arbitrary mathematical constructs, but rather are embedded in the intricate biology of cancer [25–27]. cfDNA fragmentation patterns are not uniformly distributed across the genome. These patterns are postulated to be influenced by chromatin organization, offering valuable insights into nucleosome positioning and gene expression [26]. The observed differential distribution of fragment length patterns in several chromosomes, particularly in chromosomes known to be frequently mutated in UCEC, suggests that the FSD fragmentomics features used in the classifier may reflect the degree of openness of chromosomal DNA. The pathway enrichment analysis further revealed the involvement of cancer-related pathways and immune response in UCEC patients, providing insights into the molecular mechanisms associated with the disease. The pathway enrichment analysis of differential NF features further revealed the involvement of cancer-related pathways and immune response in UCEC patients. The pathways, such as transcriptional misregulation in cancer, highlight the aberrant control of gene expression central to tumor development. Viral carcinogenesis underscores the role of viral infections, particularly HPV, in genomic instability, while chemical carcinogenesis points to the impact of environmental factors on DNA integrity. Lastly, Th17 cell differentiation emphasizes the influence of the immune system in the tumor microenvironment. Collectively, these pathways provide a comprehensive view of the molecular mechanisms driving UCEC, suggesting potential therapeutic targets and offering insights into the persistent nature of these pathogenic processes across all stages of the disease. The correlation between cancer scores and advancing stages of UCEC and tumor fraction further supports the clinical relevance of the classifier in capturing disease progression.

The analysis of preanalytical and physiological variables revealed the robustness and reliability of the classifier under various conditions. The reproducibility of results in repeated blood collections and the consistency of outcomes regardless of transportation conditions or physiological states highlight the stability of the classifier’s performance. These findings are important for the practical implementation of the classifier in clinical settings (Fig. 4).

In this study, the test was designed to target for an average sequencing depth of 5X with the consideration of cost-effectiveness and data uniformity in practical applications. Inevitably, technical variations during the sequencing process can lead to some samples achieving coverage that exceeds our target of 5 × . To ensure uniformity across all samples and to ensure that the model predicts based on genetic data rather than on an artifact of the sequencing process (i.e. variation in sequencing coverage)., we chose to down-sample any sample with a coverage higher than 5 × to a fixed depth of 5 × . Notably, based on our analysis (Fig. S3C), models trained with data at varying raw sequencing depths did not show improved performance at higher depths. It is important to highlight that our established threshold for down-sampling was 5 × , yet our analyses suggest the potential for obtaining reliable results even at sequencing depths below this threshold. This underlines the robustness of our findings against variations in sequencing depth, a factor that is of critical importance for the practical application of these methods in clinical settings. Specifically, while individual models such as FSD and NF show variability and diminished performance at reduced sequencing depths, our ensemble model exhibits a remarkable resilience, consistently maintaining reliable detection. The resilience of the ensemble model likely stems from its ability to compensate for the reduced informational content that comes with lower sequencing depths, thus ensuring stable performance.

We employed a previously established test [22] to estimate the potential clinical advantage our model could provide. According to these estimates, our approach could increase the detection of early-stage UCEC, potentially improving the 5-year survival rate from 84 to 95% when compared to the detection rates of advanced-stage disease under standard care (Additional file 1: Fig. S11). However, these findings are speculative and based on mathematical modeling rather than clinical outcomes. The study acknowledges specific limitations that must be considered. The sample size, while adequate for initial exploration, is relatively modest and might not fully represent the efficacy of our model. Consequently, the impact of our model on patient outcomes requires thorough validation in a larger and more diverse clinical setting. To address potential batch effects stemming from variations in sample processing and sequencing, we established an independent validation set based on the timing of patient enrollment and sample collection. This measure was critical to reduce confounding influences and to ensure that our model is trained and validated on temporally distinct data sets, which more accurately mirrors the variability present in clinical environments. A multi-site study design would be ideal for future research to provide a more varied and comprehensive assessment of our model’s performance.

Therefore, our findings should primarily be interpreted as a proof of concept, highlighting the importance of further research to validate and potentially expand the utility of our model across different clinical contexts. Prospective clinical trials are needed to ascertain if the early detection strengths of our model correlate with a significant improvement in patient survival. These trials would also offer valuable insights into the cost-effectiveness, practicality, and overall enhancement of patient care that our model could bring about.

## Conclusions

Summary, the results of our study underscore the promising performance and clinical potential of our classifier for the detection of UCEC. The high predictive accuracy, sensitivity in detecting early-stage disease, robustness against preanalytical and physiological variations, and the biological relevance of the fragmentomics patterns collectively affirm the classifier’s potential as a non-invasive tool for UCEC screening and diagnosis. Our results may initiate further research and potentially contribute to the advancements in the early diagnosis and improved prognosis of UCEC patients.

### Supplementary Information

Additional file 1: Fig S1- Patient enrollment flow chart; Fig S2 - Training ROC curve of base models constructed upon individual features; Fig S3 – Model performance on validation cohort with lower sequencing coverage depth; Fig S4 -  Model Performance Evaluation through Repeated Random Partitioning of the Cohort; Fig S5 - Cancer scores of 47 hysteromyoma patients; Fig S6 - Tumor fraction with stage and score; Fig S7 - Boxplot comparing cancer scores in samples with different grade and MSI status; Fig S8 - Heatmap of features; Fig S9 – Biological significance of features; Fig S10 - Fragment length distribution; Fig S11-Stage shift analysis.

Additional file 2. Sample sequencing quality metric.

Additional file 3. Features using for model.

Additional file 4. Detailed patient characteristic.

Additional file 5. 104 NF features related genes that displayed distinct characteristics in UCEC patients versus healthy controls.

## Data Availability

All raw sequencing data have been deposited in the National Genomics Data Center (NGDC) under the accession code: HRA006632. The raw sequencing data contain information unique to individuals and are available under controlled access. Access to the data can be requested by completing the application form via GSA-Human System and is granted by the corresponding Data Access Committee. Additional guidance can be found at the GSA-Human System website (https://ngdc.cncb.ac.cn/gsa-human/document/GSA-Human_Request_Guide_for_Users_us.pdf).

## Abbreviations

AUC: Area under the curve
cfDNA: Cell-free DNA
CNV: Copy number variation
ctDNA: Circulating tumor DNA
DELFI: Fragments for Early Interception
DL: Deep Learning
DRF: Distributed Random Forest
FSD: Fragment size distribution
GBM: Gradient Boosting Machine
GLM: Generalized Linear Model
NF: Nucleosome footprint
PP: A Positive Percent Agreement
TVU: Transvaginal ultrasonography
UCEC: Uterine corpus endometrial carcinoma
WGS: Whole genome sequencing
