# Supplementary material for: Early detection of uterine corpus endometrial carcinoma utilizing plasma cfDNA fragmentomics
Source: BMC Med. 2024 Jul 29;22:310. doi: 10.1186/s12916-024-03531-8 (PMC11288124; doi:10.1186/s12916-024-03531-8)
Supplement: Supplementary file 1 — Additional file 1: Fig S1- Patient enrollment flow chart; Fig S2 - Training ROC curve of base models constructed upon individual features; Fig S3 – Model performance on validation cohort with lower sequencing coverage depth; Fig S4 - Model Performance Evaluation through Repeated Random Partitioning of the Cohort; Fig S5 - Cancer scores of 47 hysteromyoma patients; Fig S6 - Tumor fraction with stage and score; Fig S7 - Boxplot comparing cancer scores in samples with different grade and MSI status; Fig S8 - Heatmap of features; Fig S9 – Biological significance of features; Fig S10 - Fragment length distribution; Fig S11-Stage shift analysis. [file 12916_2024_3531_MOESM1_ESM.pdf]

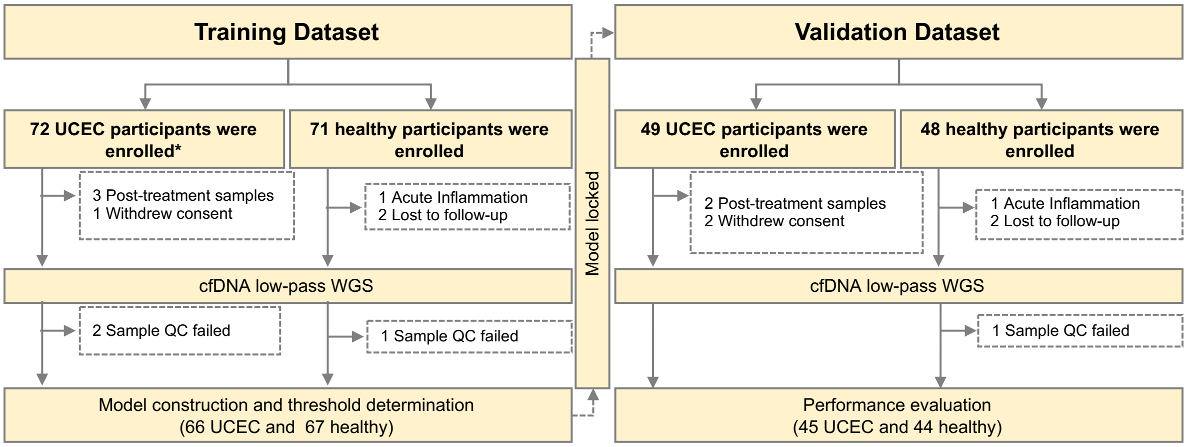

Fig S1 Patient enrollment flow chart

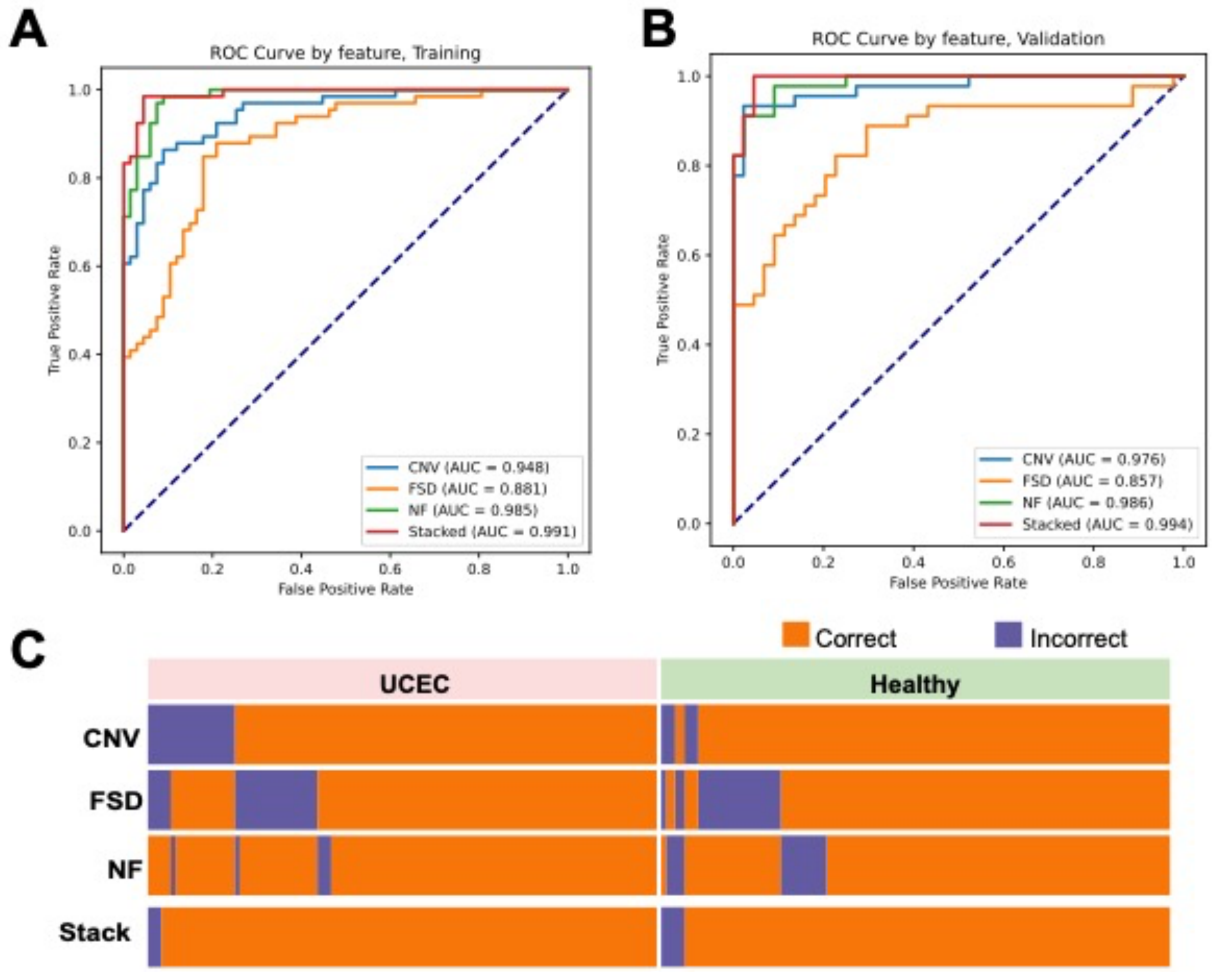

Fig S2 A-B Training ROC curve of base models constructed upon individual features (CNV, FSD and NF) and their ensemble stack model for training (**A**) and validation (**B**) cohort. **C Prediction agreement with true labels for each case under three single-feature model and the ensemble stacked model**

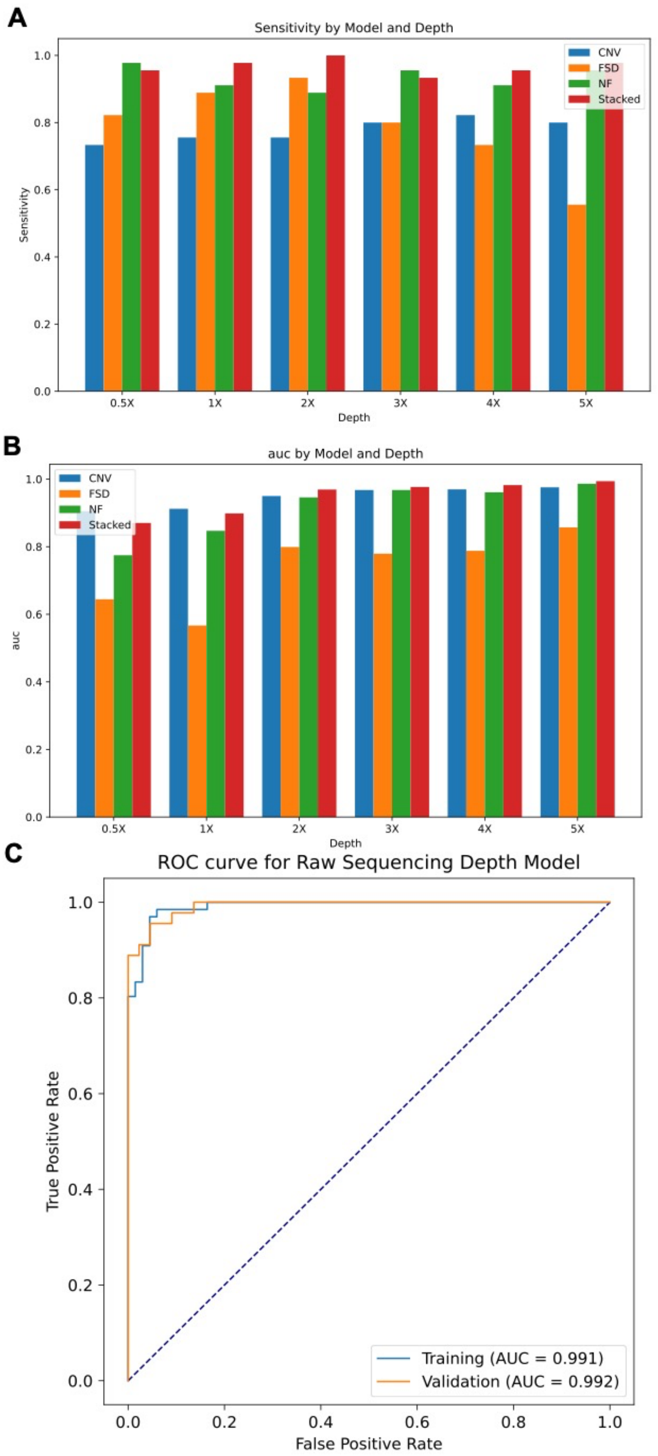

Fig S3 Sensitivity (**A**) and AUC (**B**) of the model on validation cohort with lower sequencing coverage depth. **C** ROC curve of model constructed using raw sequencing depth data.

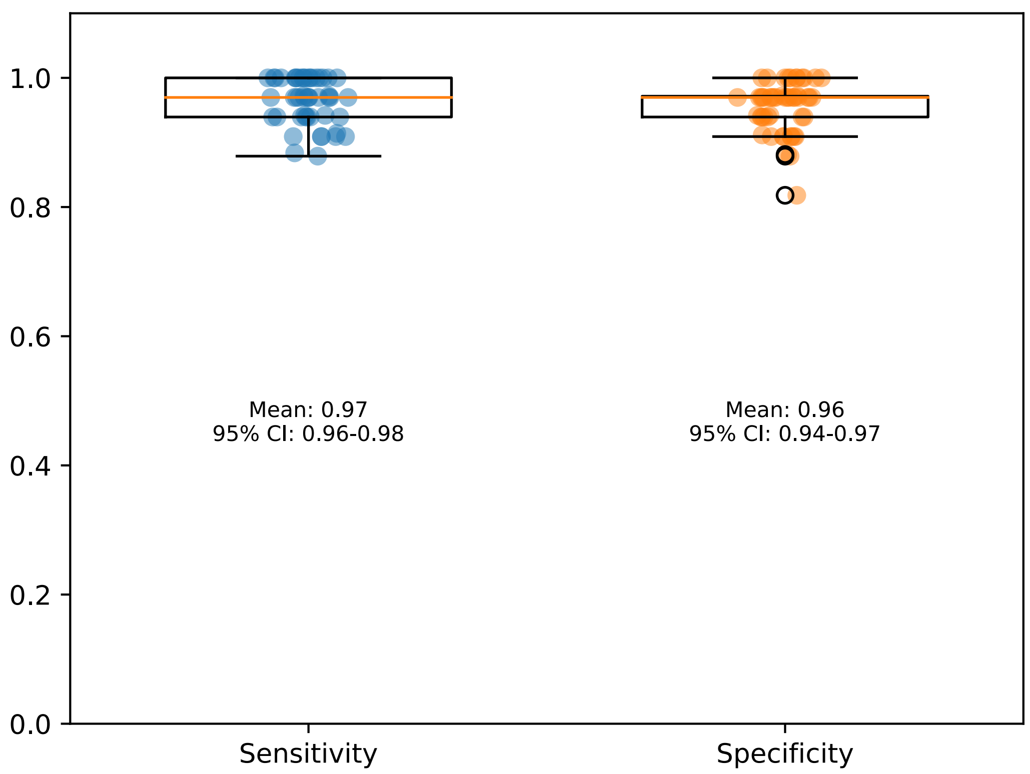

Fig S4 Model Performance Evaluation through Repeated Random Partitioning of the Cohort

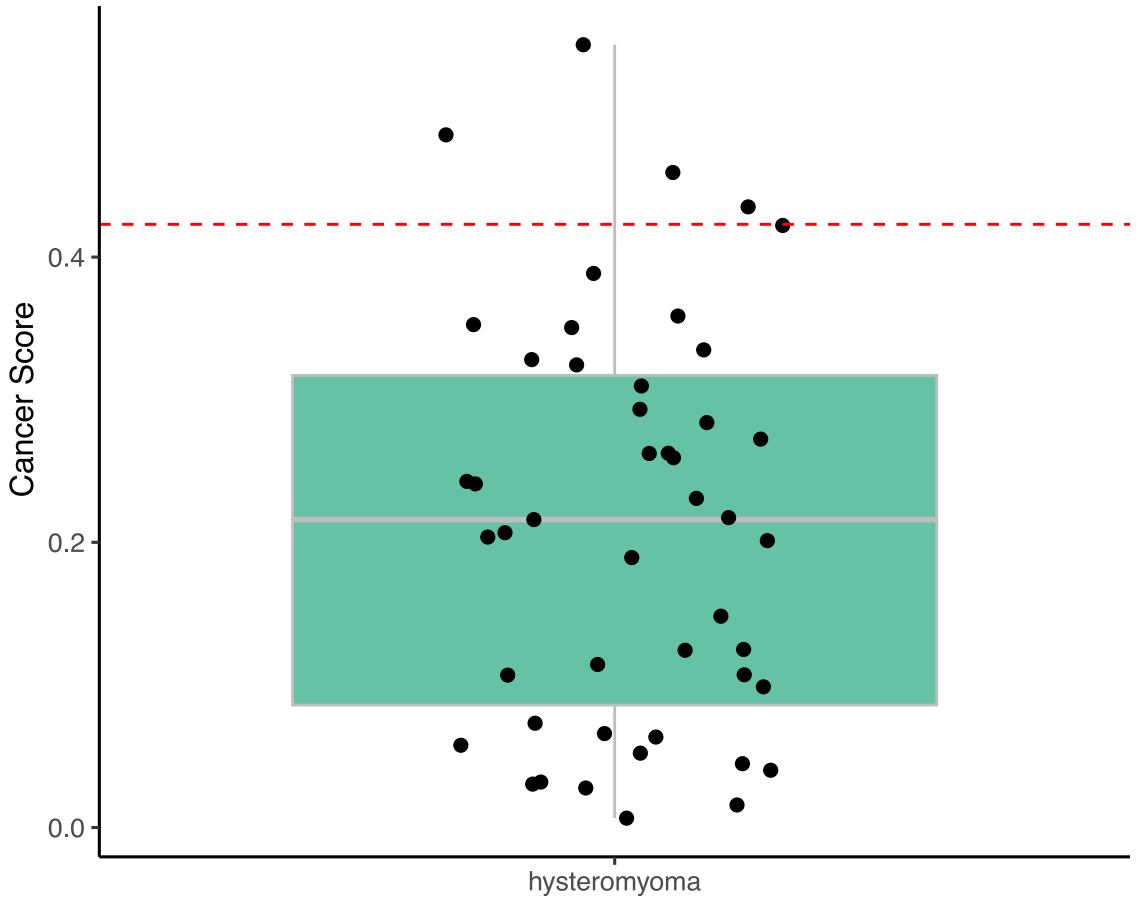

Fig S5 Cancer scores of 47 hysteromyoma patients

A

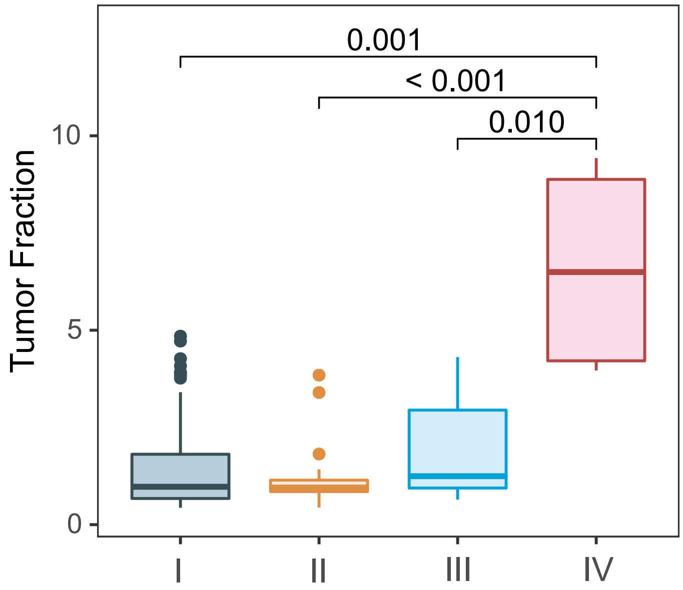

B

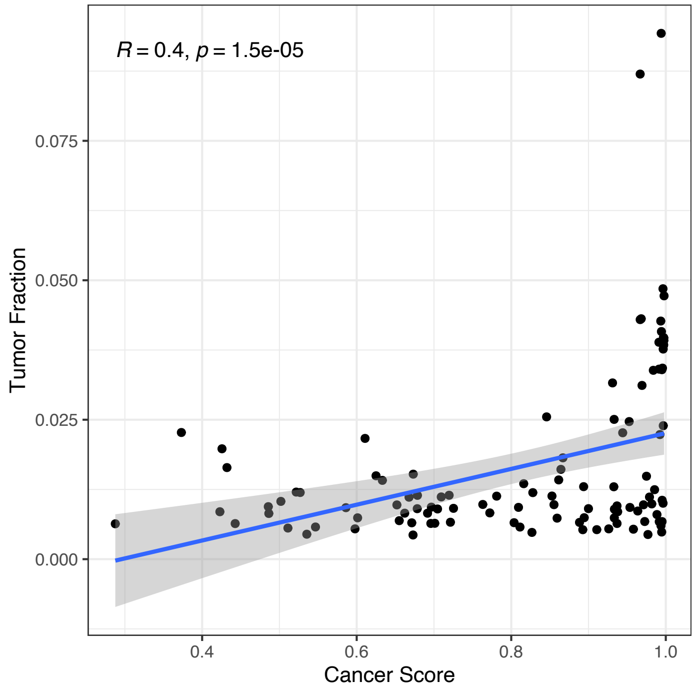

Fig S6 **A** The correlation of tumor fraction and stage. **B** Scatter plot illustrating the positive correlation between the cancer scores and the tumor fraction.

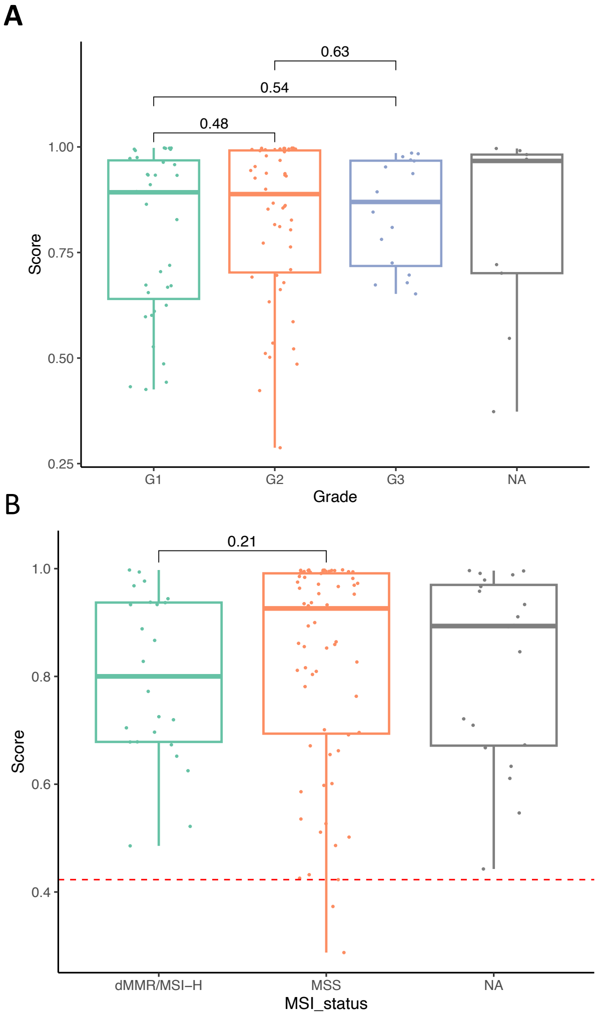

Fig S7. Boxplot comparing cancer scores in samples with different grade (A) and MSI status (B)

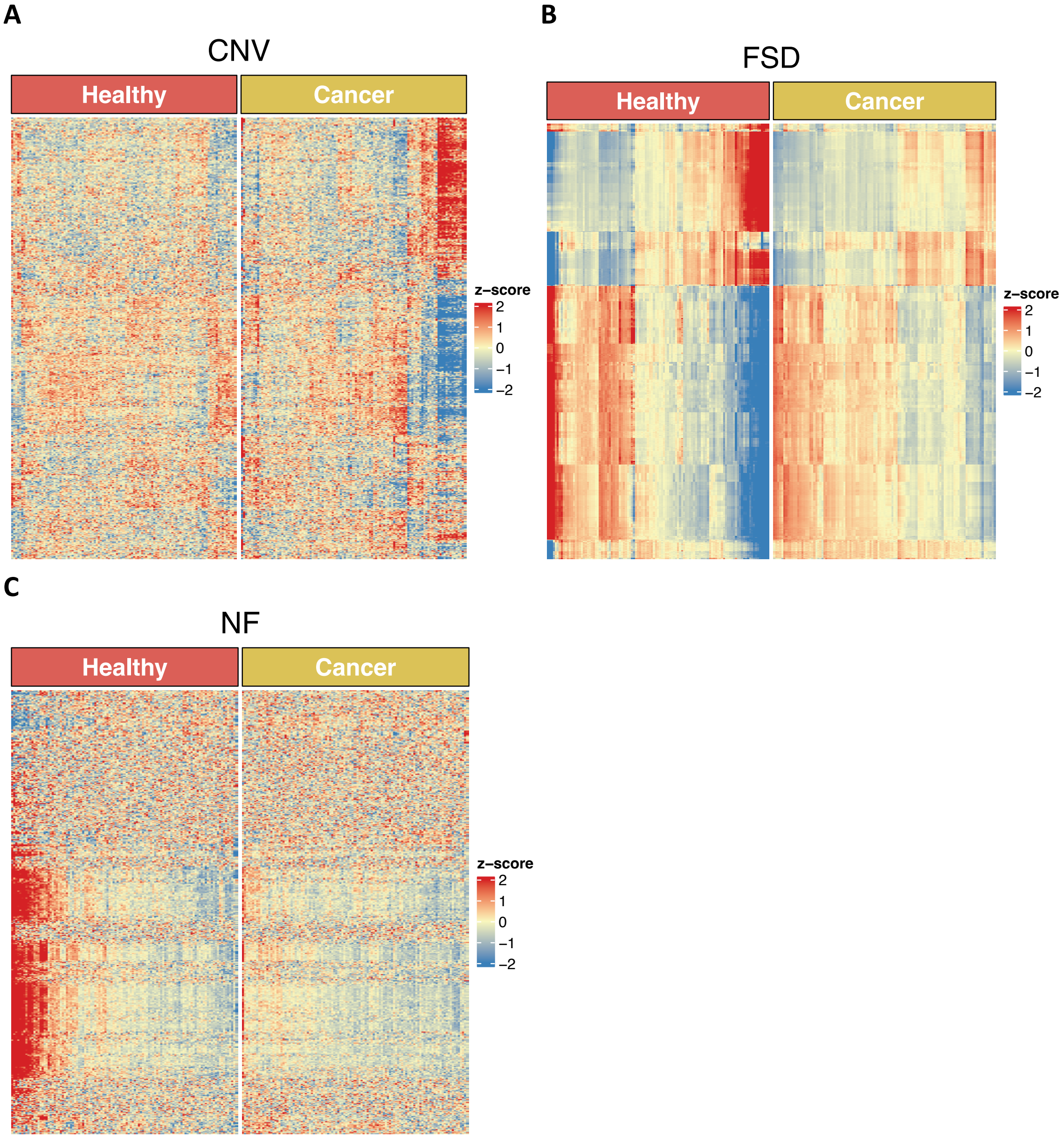

Fig S8. Heatmap showed CNV(A), FSD(B) and NF(C) features between UCEC patients and healthy control.

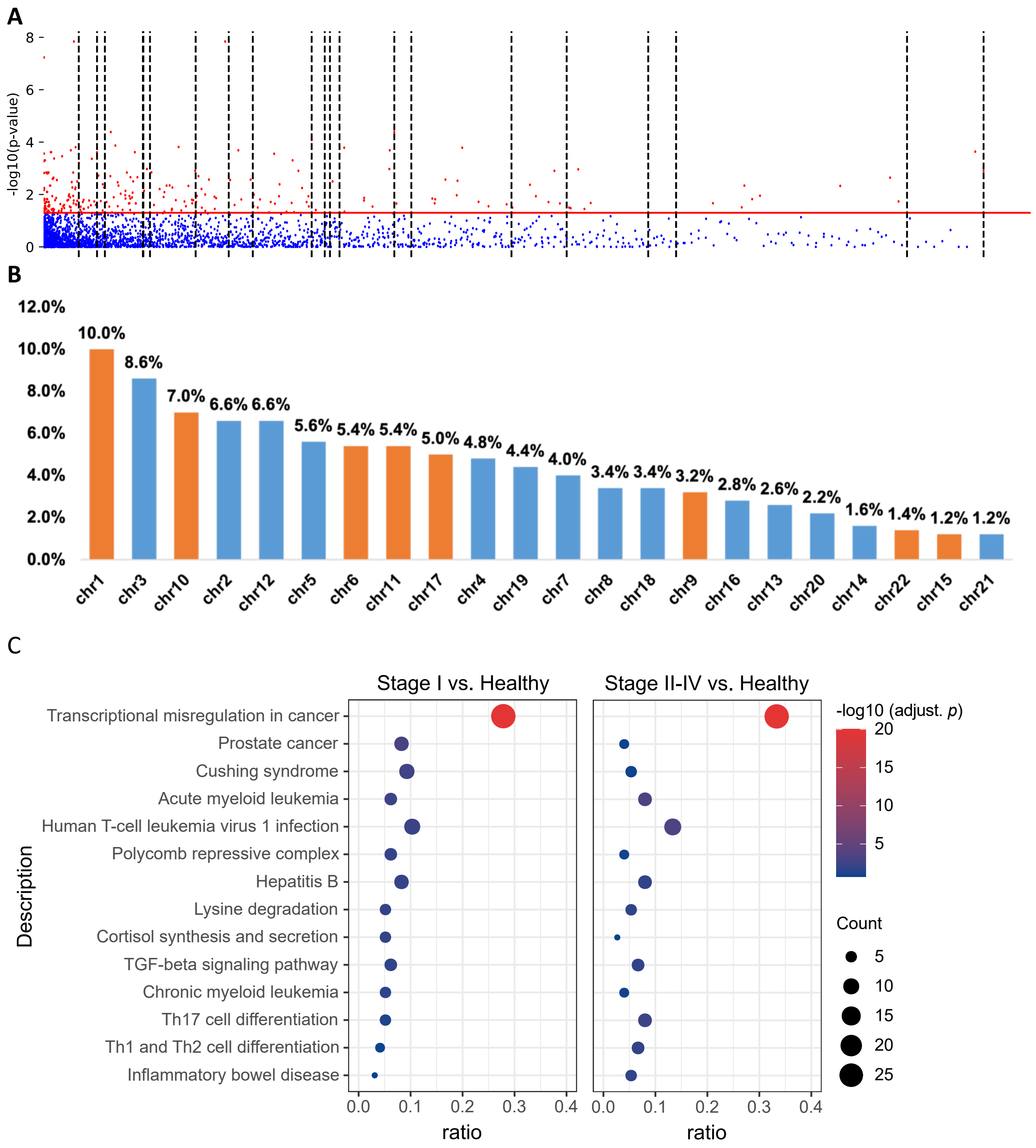

Fig S9. A CNV feature difference between UCEC patients and healthy controls. Red dots indicated a significant adjusted p-value. **B** The mutation frequency of each chromosome in TCGA-UCEC database. **C** KEGG pathway enrichment analysis of genes corresponding to NF features that displayed distinct characteristics in early stage and late stage UCEC patients versus healthy controls.

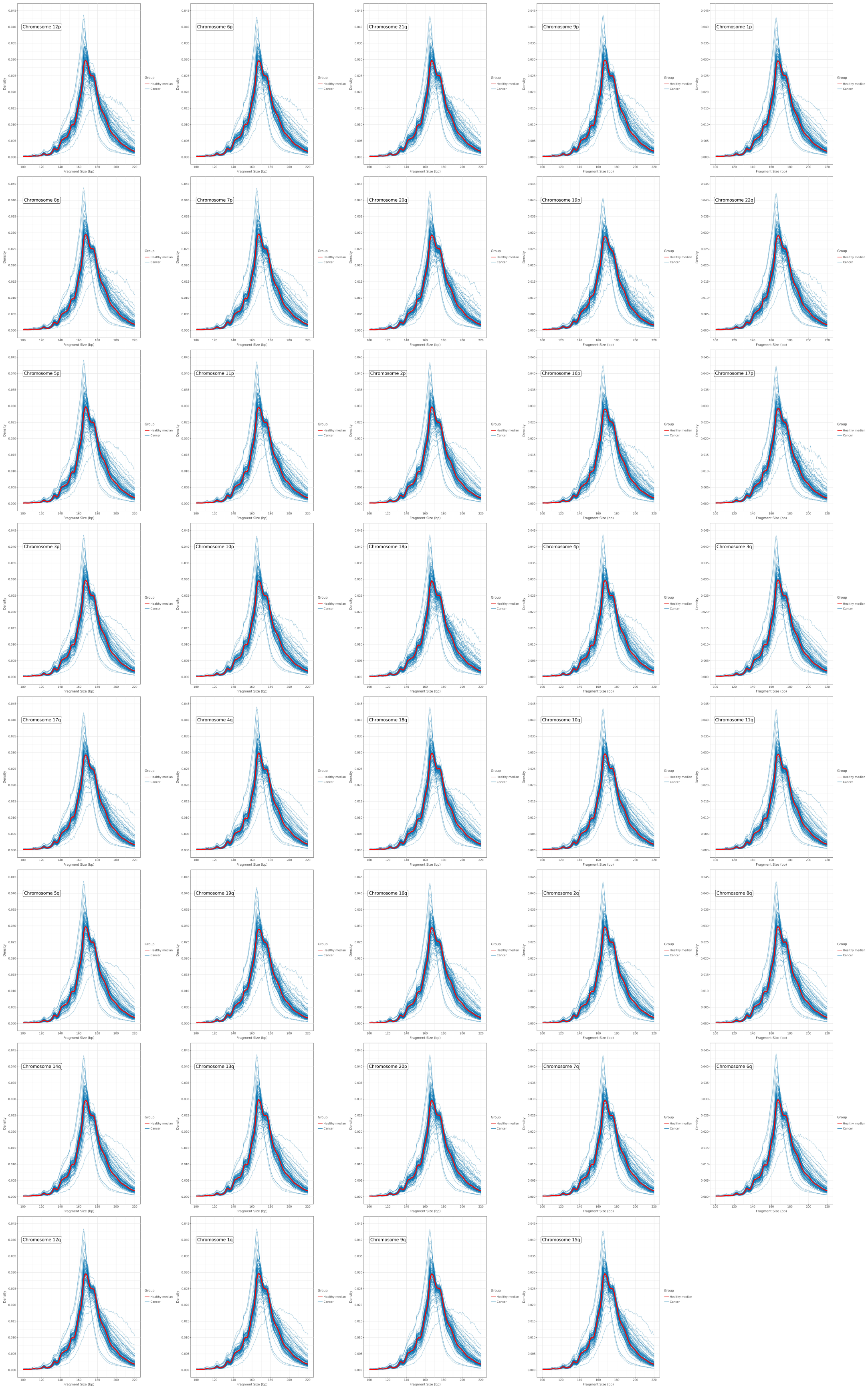
Chromosome 12p
Chromosome 6p
Chromosome 21q
Chromosome 9p
Chromosome 1p
Chromosome 8p
Chromosome 7p
Chromosome 20q
Chromosome 19p
Chromosome 22q
Chromosome 5p
Chromosome 11p
Chromosome 2p
Chromosome 16p
Chromosome 17p
Chromosome 3p
Chromosome 10p
Chromosome 18p
Chromosome 4p
Chromosome 3q
Chromosome 17q
Chromosome 4q
Chromosome 18q
Chromosome 10q
Chromosome 11q
Chromosome 5q
Chromosome 19q
Chromosome 16q
Chromosome 2q
Chromosome 8q
Chromosome 14q
Chromosome 13q
Chromosome 20p
Chromosome 7q
Chromosome 6q
Chromosome 12q
Chromosome 1q
Chromosome 9q
Chromosome 15q
Density
Fragment Size (bp)
Group
Healthy median
Cancer

Fig S10. Fragment length distribution between the UCEC and healthy donors across each chromosome arms.

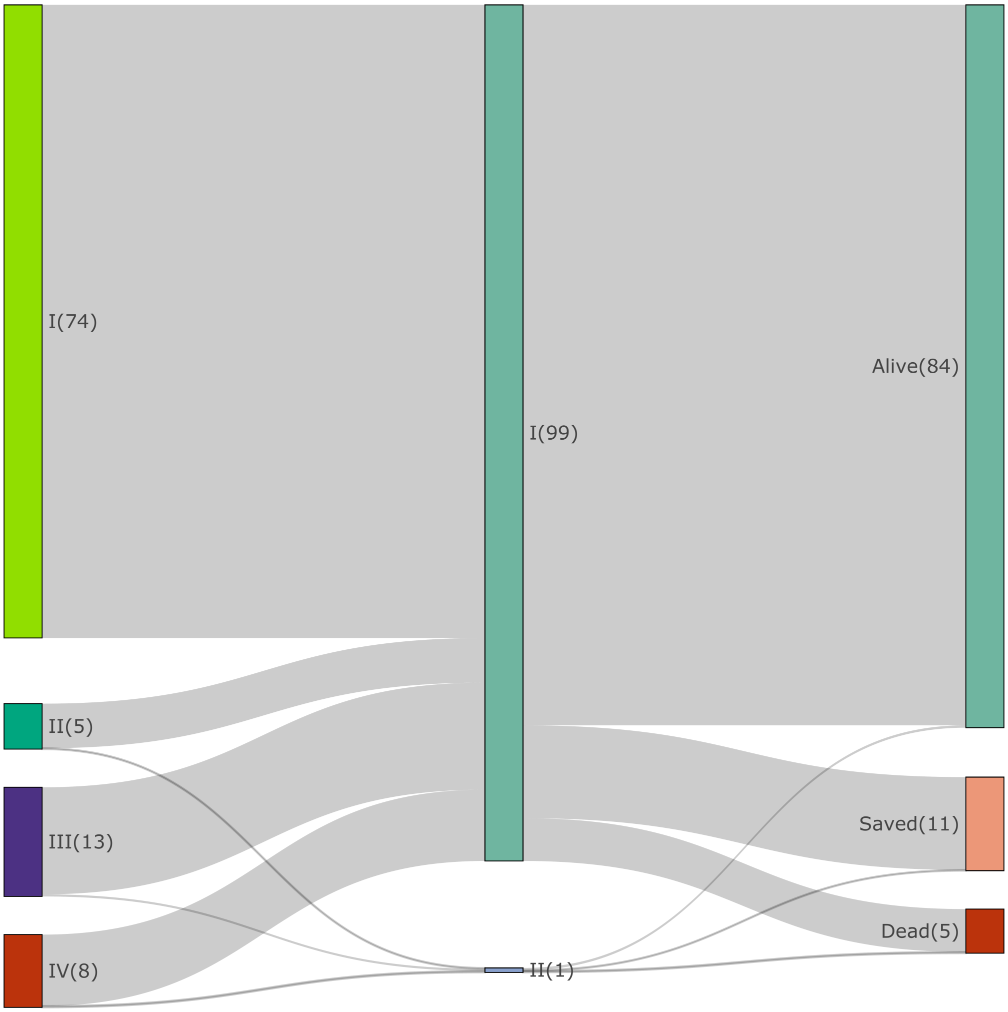

Fig S11 Stage shift analysis by the classifier in a real-world setting among the Chinese population. Sankey plot demonstrating the proportion of patients at each cancer stage at diagnosis, with and without the interception of the classifier, along with potential outcomes in real world.
